# Confirmatory trial of non-amputative digit preservation surgery for subungual melanoma: Japan Clinical Oncology Group study (JCOG1602, J-NAIL study protocol)

**DOI:** 10.1186/s12885-019-6248-2

**Published:** 2019-10-25

**Authors:** Kiyo Tanaka, Yasuhiro Nakamura, Tomonori Mizutani, Taro Shibata, Arata Tsutsumida, Haruhiko Fukuda, Shigeto Matsushita, Megumi Aoki, Kenjiro Namikawa, Shuichi Ohe, Satoshi Fukushima, Naoya Yamazaki, on behalf of the Dermatologic Oncology Group of the Japan Clinical Oncology Group

**Affiliations:** 1 JCOG Data Center/Operations Office, National Cancer Center Hospital, 5-1-1 Tsukiji, Chuo-ku, Tokyo, 104-0045 Japan; 2 Department of Skin Oncology/Dermatology, Saitama Medical University International Medical Center, 1397-1 Yamane, Hidaka, Saitama, 350-1298 Japan; 3 Department of Dermatologic Oncology, National Cancer Center Hospital, 5-1-1 Tsukiji, Chuo-ku, Tokyo, 104-0045 Japan; 4 Department of Cutaneous Oncology, Cancer Institute Hospital, 3-8-31 Ariake, Koto-ku, Tokyo, 135-8550 Japan; 5 Department of Dermato-Oncology/Dermatology, National Hospital Organization Kagoshima Medical Center, 8-1 Kagoshima Shiroyama-cho, Kagoshima, 892-0853 Japan; 6 Department of Dermatologic Oncology, Osaka International Cancer Institute, 3-1-69 Otemae, Osaka Chuo-ku, Osaka, 541-8567 Japan; 7 Department of Dermatology and Plastic Surgery, Faculty of Life Sciences, Kumamoto University, 2-39-1 Kurokami, Kumamoto Chuo-ku, Kumamoto, 860-8555 Japan

**Keywords:** Subungual melanoma, Non-amputative digit preservation surgery, Amputation, Single arm confirmatory trial, Nonrandomized trial

## Abstract

**Background:**

Amputation is the standard of care even for early-stage subungual melanomas (SUMs), known as nail apparatus melanoma, because the nail bed and nail matrix are close to the distal phalanx. However, a recent study demonstrated that not all patients with SUMs had histologic invasion of the underlying distal phalanx. As most SUMs occur in the thumb or big toe, amputation of either the thumb or big toe substantially interferes with activities of daily living, including poor cosmesis, loss of function, and phantom pain. Non-amputative digit preservation surgery can thus be applied in such cases without compromising patient prognosis.

**Methods:**

We are conducting a multi-institutional single-arm trial to confirm the safety and efficacy of non-amputative digit preservation surgery. We will compare our results with those reported in the Japanese Melanoma Study, in which patients underwent amputation for SUMs as a traditional standard of care. Patients aged between 20 and 80 years with stage I, II, or III without evidence of tumor invasion to the underlying distal phalanx on preoperative radiograph are included in the study. The primary endpoint is major relapse-free survival (major RFS), which does not include local recurrence as an event; secondary endpoints include overall survival, digit-preservation survival, relapse-free survival, local relapse-free survival, partial relapse-free survival, and incidence of adverse events. A total of 85 patients from 21 Japanese institutions will be recruited within 5.5 years, and the follow-up period will last at least 5 years. The Japan Clinical Oncology Group Protocol Review Committee approved this study protocol in August 2017, and patient enrollment began in November 2017. Ethical approval was obtained from each institution’s Institutional Review Board prior to patient enrollment.

**Discussion:**

This is the first prospective trial to confirm the safety and efficacy of non-amputative digit preservation surgery for SUM without distant metastasis or bony invasion. The results of this trial could provide evidence to support this less-invasive surgery as a new standard of care to preserve adequately functioning digits.

**Trial registration:**

Registry number: UMIN000029997. Date of Registration: 16/Nov/2017. Date of First Participant Enrollment: 12/Dec/2017.

## Background

The incidence of melanoma differs by ethnicity but has been increasing recently among all populations. The incidence of melanoma per 100,000 people in 1999 was 21.3 in Caucasians and 1.3 in Asians, but in 2008, it reached 26.5 in Caucasians and 1.6 in Asians [1]. The frequency of the sites involved also differs by ethnicity. Although melanoma in Asians and Africans is generally much rarer than in Caucasians, the incidence of subungual melanoma (SUM), also known as nail apparatus melanoma, is ten-fold higher among Asians than among Caucasians [2–7]. The most common sites of SUM are the thumb and big toe [8]. Given the rising incidence of melanoma, the number of Japanese patients with SUM of the thumb or big toe is increasing.

Amputation of the digit has been the traditional standard surgical procedure for SUMs because the nail bed and nail matrix are close to the distal phalanx [9–11]. However, amputation of either the thumb or big toe substantially interferes with activities of daily living. Adverse events associated with digital bone resection include poor cosmesis, loss of function, formation of clavus or tylosis on the sole, and phantom pain. Amputation of the thumb is particularly distressing. Smock et al. [12] noted that 40% of patients report loss of function that interferes with normal activities.

Meanwhile, the excision margin for SUM remains controversial. Although amputation around the distal interphalangeal joint has been advised, more proximal amputation has not improved patient outcomes [13]. The prognosis for patients with SUM does not depend on the extent of amputation but rather on the time from initial diagnosis to surgery. Non-amputative digit preservation surgery has been performed for SUMs that are either in situ or ≤ 0.5-mm thick [14]. Furthermore, a recent study indicated that not all patients with SUM had histologic invasion in the underlying distal phalanx [15]. Very little data on patient prognosis after non-amputative digit preservation surgery are available, but several case reports or case series of patients with invasive SUM treated with non-amputative digit preservation surgery found a low incidence of recurrence and favorable clinical prognosis [12, 16–18]. Based on the results from these studies, non-amputative digit preservation surgery appears to be useful in such patients without compromising their prognosis.

Therefore, we designed a multi-institutional single-arm trial to confirm the safety and efficacy of non-amputative digit preservation surgery for SUM without distant metastasis or bony invasion, which could support a new standard of less-invasive surgery to preserve adequately functioning digits. Because it is difficult to collect a sufficient number of patients with SUM for a randomized study, this trial is a single-arm trial rather than a randomized controlled trial. However, reliable data from the Japanese Melanoma Study (unpublished data) are available for comparison as historical controls.

The Japan Clinical Oncology Group (JCOG) Protocol Review Committee approved this study protocol in August 2017, and patient enrollment began in November 2017. Approval was obtained from each institution’s Institutional Review Board prior to patient recruitment.

## Methods/design

### Study design and endpoints

JCOG1602 (J-NAIL study) is a multicenter, single-arm, confirmatory trial to evaluate the safety and efficacy of non-amputative digit preservation surgery for patients with SUM without distant metastasis or bony invasion.

The primary endpoint is major relapse-free survival (RFS), defined as the number of days from patient registration to major relapse (defined below) or death from any cause, censored at the last day the patient is alive without any evidence of major relapse. Major relapse is defined as satellite or in-transit metastases or metastasis to regional or distant lymph nodes or to distant organs, but it does not include local relapse (Table 1).

**Table 1:** Definition of endpoints

Endpoint	Event								Censoring
	Death from any cause	Local recurrence	Satellite metastasis	in-transit metastasis	Regional LN metastasis	Distant LN metastasis	Distant organ metastasis	Amputation for any cause	
Major relapse-free survival	+	–	+	+	+	+	+	–	Last day the patient is alive
Local relapse-free survival	+	+	–	–	–	–	–	–	
Digit-preservation survival	+	–	–	–	–	–	–	+	
Relapse-free survival	+	+	+	+	+	+	+	–	
Overall survival	+	–	–	–	–	–	–	–	
Partial relapse-free survival	+	–	–	+	+	+	+	–	

*LN* Lymph node

The secondary endpoints are local RFS, digit-preservation survival, RFS, overall survival (OS), partial RFS, and adverse events (Table 1). Local RFS is defined as the number of days from registration to local relapse. Digit-preservation survival is defined as the number of days from registration to digit amputation with any cause (e.g., performed as a salvage surgery for local relapse with bony invasion). RFS is defined as the number of days from registration to local or major relapse. OS is defined as the number of days from registration to death from any cause. Partial RFS is defined as the number of days from registration to partial relapse, which is defined as the development of in-transit metastases or metastasis to regional or distant lymph nodes or to distant organs. All endpoints except for adverse events also include death from any cause as an event and are censored at the last day the patient is alive without relevant events (Table 1).

### Study population and eligibility criteria

Patients aged between 20 and 80 years with stage I, II, or III disease (American Joint Committee on Cancer TNM Staging System for Melanoma 7th edition, 2009 [19]) and without evidence of tumor invasion to the underlying distal phalanx on preoperative radiograph have been included in the study. Patients with satellite or in-transit metastases have been excluded. Detailed inclusion and exclusion criteria are shown in Table 2.

**Table 2:** Inclusion and exclusion criteria for J-NAIL study

Inclusion criteria	
1) Invasive subungual melanoma that is clearly diagnosable by clinical and dermoscopic findings alone or histologically diagnosable by a biopsy specimen.	
2) Not suspected to be melanoma in situ by clinical findings or dermoscopy images.	
3) Tumor circumference, including Hutchinson spread, ≤80% of the perimeter of the phalanx.	
4) No satellite or in-transit metastases.	
5) No tumor invasion to the distal phalanx cortical bone by X-ray examination.	
6) No distant metastases on whole-body computed tomography.	
7) No unresectable lymph node metastases.	
8) Age ≤ 20 and ≤ 80 years.	
9) Eastern Cooperative Oncology Group (ECOG) performance status of 0 to 2.	
10) No previous treatment including surgery, chemotherapy, radiotherapy, or immunotherapy for primary cancer.	
11) Adequate organ and marrow function as defined below within 28 days prior to registration:	
a) White blood cell count ≥2500 /mm^3^	
b) Hemoglobin ≥9.0 g/dL	
c) Platelet count ≤80,000 /mm^3^	
d) Total bilirubin ≤2.0 mg/dL	
e) Aspartate aminotransferase ≤150 IU/L	
f) Alanine aminotransferase ≤150 IU/L	
Exclusion criteria	
1) Synchronous or metachronous (within 5 years) malignancy, except cancer with a 5-year relative survival rate of 95% or more, such as carcinoma in situ, intramucosal tumor, or early-stage cancer.	
2) Active infection requiring systemic therapy.	
3) Body temperature ≥ 38 °C.	
4) Women who are pregnant or nursing.	
5) Patients with severe psychiatric disease.	
6) Patients requiring systemic steroid medication or other immunosuppressive drugs.	
7) Poorly controlled diabetes.	
8) Poorly controlled hypertension.	
9) History of unstable angina pectoris within 3 weeks or myocardial infarction within 6 months before registration.	

### Treatment

**Fig. 1:**
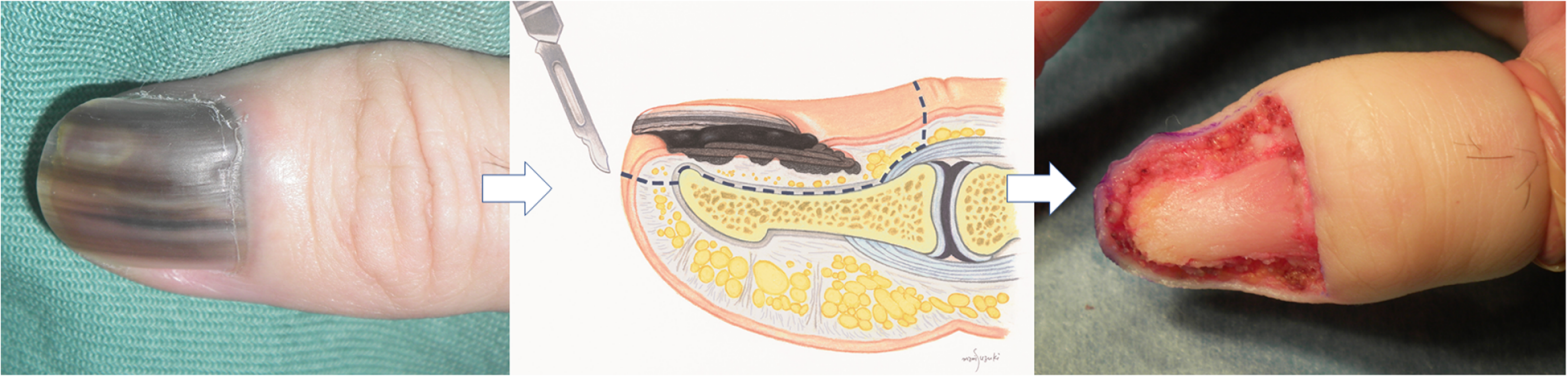
Scheme of non-amputative digit preservation surgery

**Fig. 2:**
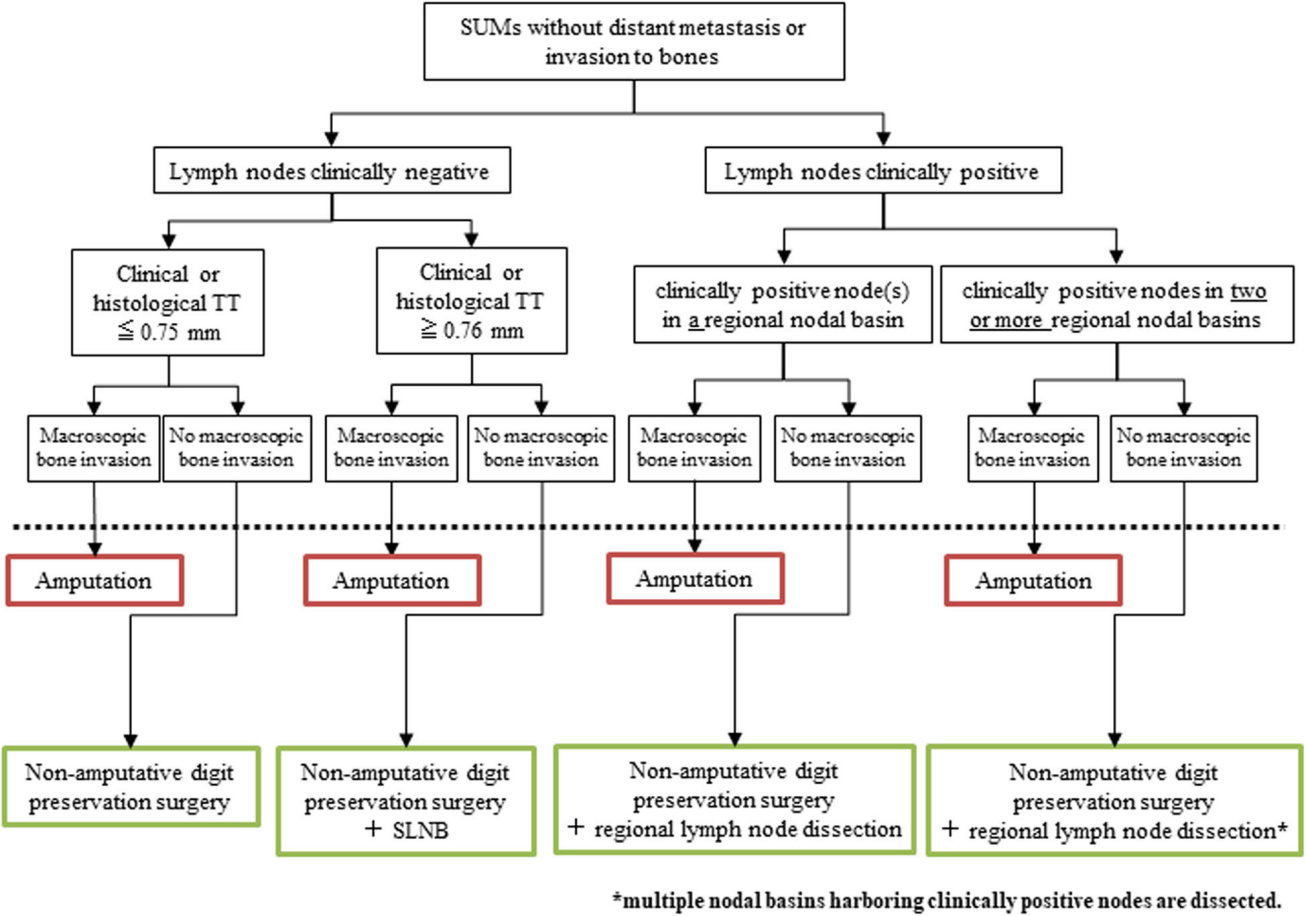
The algorithm for the JCOG1602 treatment protocol. SLNB, sentinel lymph node biopsy; SUM, subungual melanoma; TT, tumor thickness

The treatment protocol for the study is non-amputative digit preservation surgery with or without sentinel lymph node biopsy or regional lymph node dissection. Non-amputative digit preservation surgery includes en bloc resection of the tumor and nail apparatus, including the periosteum of the distal phalanx (Fig. 1). The tissue defect is tentatively covered by an artificial dermis intraoperatively. If negative deep margins cannot be obtained, digit amputation is performed at the level of either the distal interphalangeal or the metacarpophalangeal or tarsophalangeal joint as a salvage surgery. The exact treatment protocols in this study differ for each patient depending on the extent of disease. The treatment protocol is based on an algorithm (Fig. 2) that includes the presence or absence of clinical lymphadenopathy and clinical or histologic tumor thickness. Sentinel lymph node biopsy is performed in patients with tumors that measure 0.76 mm in thickness or greater and without regional lymph node enlargement. Lymphoscintigraphy using radioisotope and intraoperative hand-held gamma-probe and vital blue-dye are utilized to detect sentinel lymph nodes. Regional lymph node dissection is performed for patients with regional lymph node enlargement.

Treatment after the completion or termination of protocol surgery is not regulated, but additional surgery is recommended according to the pathology results, for example, reconstruction of a tissue defect, sentinel lymph node biopsy, regional lymph node dissection, skin resection, or amputation.

### Follow-up

All registered patients will be followed for at least 5 years after recruitment is completed. Palpation plus inspection and dermoscopy will be performed at least every 3 months for 5 years after surgery and every 6 months thereafter. Enhanced computed tomography of the neck, chest, abdomen, and pelvis will be evaluated at least every 6 months for 5 years after surgery and every year thereafter. If a patient exhibits symptoms that are indicative of central nervous system metastasis, magnetic resonance imaging of the brain will be performed.

### Historical control

The Japanese Skin Cancer Society Prognosis Survey Committee has recorded the staging prognosis of 141 patients with SUM since 2005 (Japanese Melanoma Study). The data include patients with bone invasion, which was confirmed by postoperative pathology. Therefore, the Japanese Melanoma Study data, with the exclusion of patients with bone invasion, were used as a historical control. In the historical control, the 5-year OS rates of patients with stage I, II, and III disease were 100, 83.7, and 56.2%, respectively. The 5-year RFS rates of patients with stage I, II, and III disease were 100, 81.1, and 44.7%, respectively.

### Statistical consideration

#### Sample size calculation and statistical analysis

Primary analysis is to be carried out 5 years after recruitment is complete. In a previous study (Japanese Melanoma Study) conducted by the Japanese Skin Cancer Society, the 5-year major RFS was 73%. We anticipate that the 5-year major RFS for this study will be 77%, considering the strict eligibility criteria in this study, JCOG1602, compared with historical controls. We consider a threshold of 67% to indicate a benefit in favor of non-amputative digit preservation surgery. If the lower limit of the 80% confidence interval of the 5-year major RFS estimated by the Kaplan-Meier method is greater than 67%, non-amputative digit preservation surgery will be concluded to be the new standard therapy for SUM without distant metastasis or bony invasion. The required sample size is 82 patients, assuming an expected 5-year major RFS of 77% and a threshold of 67% with a one-sided alpha of 0.1 and a power of 0.7. The total sample size was therefore set at 85 patients, allowing for some loss to follow-up. All statistical analyses will be conducted at the JCOG Data Center.

#### Registration and data entry

After confirming eligibility, registration with the JCOG Data Center is performed by a web-based system.

#### Interim analysis and monitoring

We do not plan to conduct formal interim analysis for efficacy because sufficient information will not be available to judge study termination during the study period.

In terms of safety, the trial will be terminated if 2 treatment-related deaths or 23 major relapse events occur. The JCOG Data Center and Study Coordinator will conduct central monitoring and will issue a monitoring report every 6 months to evaluate study progress and improve data integrity and patient safety. For quality assurance, site visit audits will be performed by the JCOG Audit Committee.

## Discussion

JCOG1602 (J-NAIL) is a multi-institutional single-arm trial to confirm the safety and efficacy of non-amputative digit preservation surgery for patients with SUM without distant metastasis or bony invasion. Primary analysis of this trial will be performed in 2029. If the results of this clinical trial prove noninferiority regarding major relapse-free survival in comparison to historical control data on amputation surgery, non-amputative wide excision will become the new standard of care, which will provide greatly improved quality of life for patients with SUM, such as preserved digit function and cosmetic appearance.

Although this trial is not a randomized controlled trial due to the rarity of patients with SUM, because of its comparison of JCOG1602 (J-NAIL) data with robust historical control data, this study can be considered a confirmatory trial. Thus, this world’s first prospective trial will provide us with crucial clinical information concerning the safety and efficacy of less-invasive surgery for SUM patients with higher levels of evidence than ever before.

### Status of the trial

The contents of this study protocol were selected and presented at the 2018 American Society of Clinical Oncology (ASCO) Annual Meeting [20]. The results will be disseminated to the public through social media and conferences. On December 12, 2017, we enrolled the first patient at the JCOG Data Center. In total, 27 patients have been enrolled as of June 23, 2019.

## Data Availability

Not applicable.

## Abbreviations

ECOG: Eastern Cooperative Oncology Group
JCOG: the Japan Clinical Oncology Group
LN: Lymph node
OS: Overall survival
RFS: Relapse-free survival
SUM: Subungual melanoma

## References

[1] SEER data base. Available from: https://seer.cancer.gov/explorer/application.php.

[2] Hori Megumi, Matsuda Tomohiro, Shibata Akiko, Katanoda Kota, Sobue Tomotaka, Nishimoto Hiroshi (2015). Cancer incidence and incidence rates in Japan in 2009: a study of 32 population-based cancer registries for the Monitoring of Cancer Incidence in Japan (MCIJ) project. Japanese Journal of Clinical Oncology.

[3] Clark WH, Elder DE, Van Horn M (1986). The biologic forms of malignant melanoma. Hum Pathol.

[4] Curtin JA, Busam K, Pinkel D (2006). Somatic activation of KIT in distinct subtypes of melanoma. J Clin Oncol.

[5] Curtin JA, Fridlyand J, Kageshita T (2005). Distinct sets of genetic alterations in melanoma. N Engl J Med.

[6] Dawber RP, Colver GB (1991). The spectrum of malignant melanoma of the nail apparatus. Semin Dermatol.

[7] Blessing K, Kernohan NM, Park KG (1991). Subungual malignant melanoma: clinicopathological features of 100 cases. Histopathology.

[8] Haneke E (2012). Ungual melanoma –controversies in diagnosis and treatment. Dermatol Ther.

[9] Daly JM, Berlin R, Urmacher C (1987). Subungual melanoma: a 25-year review of cases. J Surg Oncol.

[10] Banfield CC, Redburn JC, Dawber RP (1998). The incidence and prognosis of nail apparatus melanoma. A retrospective study of 105 patients in four English regions. Br J Dermatol.

[11] Heaton KM, el-Naggar A, Ensign LG (1994). Surgical management and prognostic factors in patients with subungual melanoma. Ann Surg.

[12] Smock ED, Barabas AG, Geh JL (2010). Reconstruction of a thumb defect with Integra following wide local excision of a subungual melanoma. J Plast Reconstr Aesthet Surg.

[13] Moehrle M, Metzger S, Schippert W (2003). “Functional” surgery in subungual melanoma. Dermatol Surg.

[14] Sureda N, Phan A, Poulalhon N (2011). Conservative surgical management of subungual (matrix derived) melanoma: report of seven cases and literature review. Br J Dermatol.

[15] Nakamura Y, Fujisawa Y, Teramoto Y (2014). Tumor-to-bone distance of invasive subungual melanoma: an analysis of 30 cases. J Dermatol.

[16] Rayatt SS, Dancey AL, Davison PM (2007). Thumb subungual melanoma: is amputation necessary?. J Plast Reconstr Aesthet Surg.

[17] Cohen T, Busam KJ, Patel A (2008). Subungual melanoma: management considerations. Am J Surg.

[18] Nakamura Y, Ohara K, Kishi A (2015). Effects of non-amputative wide local excision on the local control and prognosis of in situ and invasive subungual melanoma. J Dermatol.

[19] Edge SB, Byrd DR, Compton CC, et al., editors. AJCC Cancer Staging Handbook. In: From the AJCC Cancer Staging Manual. 7th ed. New York: Springer-Verlag; 2010.

[20] Nakamura Yasuhiro, Tanaka Kiyo, Shibata Taro, Mizusawa Junki, Mizutani Tomonori, Fukuda Haruhiko, Tsutsumida Arata, Namikawa Kenjiro, Takahashi Akira, Fujisawa Yasuhiro, Yoshikawa Shusuke, Matsushita Shigeto, Yamazaki Naoya (2018). Confirmatory trial of non-amputative digit preservation surgery in subungual melanoma: JCOG1602 (J-NAIL study). Journal of Clinical Oncology.

